# Novel Thermosensitive-*co*-Zwitterionic Sulfobetaine Gels for Metal Ion Removal: Synthesis and Characterization

**DOI:** 10.3390/gels7040273

**Published:** 2021-12-17

**Authors:** Eva Oktavia Ningrum, Takehiko Gotoh, Wirawan Ciptonugroho, Achmad Dwitama Karisma, Elly Agustiani, Zela Marni Safitri, Muhammad Asyam Dzaky

**Affiliations:** 1 Department of Industrial Chemical Engineering, Faculty of Vocational Studies, Institut Teknologi Sepuluh Nopember, Kampus ITS Sukolilo, Surabaya 60111, Indonesia; eva-oktavia@chem-eng.its.ac.id (E.O.N.); dwitama@its.ac.id (A.D.K.); elly@chem-eng.its.ac.id (E.A.); zela.171041@mhs.its.ac.id (Z.M.S.); 10411710000059@mhs.its.ac.id (M.A.D.); 2 Department of Chemical Engineering, Graduate School of Advanced Science and Engineering, Hiroshima University, Kagamiyama 1-4-1, Higashi-Hiroshima 739-8527, Japan; 3 Chemical Engineering Department, Faculty of Engineering, Sebelas Maret University, Jalan Ir. Sutami 36A, Surakarta 57126, Indonesia; wirawan_ciptonugroho@staff.uns.ac.id

**Keywords:** adsorption, heavy metals, zwitterion

## Abstract

Zwitterionic betaine polymers are promising adsorbents for the removal of heavy metal ions from industrial effluents. Although the presence of both negative and positively charged groups imparts them the ability to simultaneously remove cations and anions, intra- and/or inter-chain interactions can significantly reduce their adsorption efficiencies. Therefore, in this study, novel gels based on crosslinked co-polymers of thermosensitive *N*-isopropylacrylamide (NIPAAM) and zwitterionic sulfobetaine *N*,*N*-dimethylacrylamido propyl ammonium propane sulfonate (DMAAPS) were synthesized, characterized, and evaluated for ion removal. Fourier-transform infrared (FTIR) and proton nuclear magnetic resonance (^1^H NMR) analyses confirmed the success of the co-polymerization of NIPAAM and DMAAPS to form poly(NIPAAM-*co*-DMAAPS). The phase transition temperature of the co-polymer increased with increasing DMAAPS content in the co-polymer, indicating temperature-dependent amphiphilic behavior, as evidenced by contact angle measurements. The ion adsorption analyses of the poly(NIPAAM-*co*-DMAAPS) gels indicated that co-polymerization increased the molecular distance and weakened the interaction between the DMAAPS-charged groups (SO_3_^−^ and N^+^), thereby increasing the ion adsorption. The results confirmed that, with a low concentration of DMAAPS in the co-polymer gels (~10%), the maximum amount of Cr^3+^ ions adsorbed onto the gel was ~58.49% of the sulfonate content in the gel.

## 1. Introduction

Water quality degradation is a critical concern worldwide. This severity is reflected in the United Nations Sustainable Development Goals (SDGs), where water quality-related concerns are assigned with SDG numbers of 6 and 14 [1]. Hypothetically, the manufacturing industries are a dominant contributor to the degradation of water quality [2], as many of them release pollutants, including heavy metals such as Pb, Cr, Cu, Cd, Ni, and Zn, to the nearby water bodies [3]. These metals may cause critical health problems when they accumulate in the human body. The Cr^3+^ ion, in particular, has a considerable negative impact on the environment due to its binding affinity toward proteins and nucleic acids [4,5].

According to the World Health Organization, heavy metals may cause several types of diseases, ranging from allergic reactions to cancer [6]. Therefore, certain threshold limits have been set to regulate the disposal of these metals. For example, the Cr(VI) concentration in the inland surface water and potable water should not exceed 0.1 mg/L and 0.05 mg/L, respectively [6,7].

Over the years, several methods have been developed to reduce the concentration of heavy metals in water, including chemical precipitation, electrochemical reduction, activated carbon adsorption, ion exchange, and reverse osmosis [7]. These methods have been proven effective in reducing Cr concentration in drinking water. However, they still exhibit some drawbacks, such as high energy requirements, incomplete metal removal, and high sludge production [7]. Adsorption is another promising and economical method. The efficacy of adsorption in reducing heavy metal concentrations highly depends on the type of adsorbent. To date, several classes of adsorbents based on their origin have been developed. For example, bio-adsorbents are obtained from algae, microbial bio-mass, or non-living bio-mass, such as bark, lignin, and crustacean shells. These types of adsorbents are often preferred from an economic perspective.

Zwitterionic polymers contain equimolar amounts of homogenous anionic and cationic groups on their chains [8]. When these oppositely charged moieties are combined, the polymers exhibit ultra-hydrophilicity while preserving an overall charge neutrality [9]. This hydrophilicity enables zwitterionic polymers to be significantly effective in non-specific protein adsorption, making these materials excellent candidates for long-term anti-fouling applications [10,11]. Zwitterionic polymers have unique structures, enabling them to exhibit various properties, such as overall charge neutrality, hydration capacity, high hydrophilicity, and anti-polyelectrolyte effects in aqueous solutions; they can also behave as solid dipole pairs and have thus attracted increasing interest from many researchers [12,13]. The anti-polyelectrolyte effect enables zwitterionic polymers to frequently undergo chain expansion as their ionic strength increases in water [14]. As one of the most recent technological advances, zwitterionic polymers have emerged as a promising class of adsorbents for heavy metal ion removal. The presence of both positive and negative charges in the polymer structure improves its metal removal efficiency and selectivity [15]. Zwitterionic polymers demonstrate good adsorption capacities for Pb^2+^ and Cu^2+^, suggesting a good prospect for commercial use on an industrial scale [16]. They do not dissolve in water if the temperature is below their upper critical solution temperature (UCST) [17]. At temperatures below the UCST, the zwitterionic polymer exists in a collapsed coil condition owing to its intra- and/or inter-chain interactions. However, at temperatures above the UCST, thermal energy can overcome these interactions [18]. The use of zwitterionic polymers as adsorbents is recognized as one of the methods to selectively adsorb ions because both anions and cations in the solution have the ability to bond with oppositely charged groups [19,20]. Moreover, zwitterionic betaine polymers can simultaneously adsorb both anions and cations in liquid waste [21].

Thermoresponsive polymers have also attracted considerable interest owing to their reversible phase-transition behavior at lower critical solution temperatures (LCSTs). To extend the applications of zwitterionic materials, thermoresponsive co-polymers containing zwitterionic monomers have been synthesized [22,23,24]. Poly(*N*-isopropylacrylamide) (poly[NIPAAM]) is a thermosensitive polymer exhibiting an LCST of 32–34 °C. The UCSTs and LCSTs of polymers containing both thermosensitive NIPAAM and sulfobetaine monomer [24] can be varied by introducing specific ions [25] or zwitterions [26,27,28,29] or by varying the temperature, type of salt, or polymer concentration [30]. NIPAAM hydrogels swell in water at temperatures below the LCST and shrink with increasing temperature. Furthermore, minor temperature changes around the LCST cause rapid swelling–de-swelling transitions [31,32,33]. Due to their unique characteristic, thermosensitive NIPAAM-based polymer adsorbents have been used to adsorb and detect various heavy metal ions, such as Pb [34,35,36], Cu [37,38,39], Ni [40], Co [41,42], and Mn [43] ions. Moreover, poly(NIPAAM)-based microgels containing metal organic frameworks (MOFs) have been employed for the adsorption/desorption of selected analytes from aqueous environments [44].

Zwitterionic polymers and gels have been synthesized and characterized for the adsorption process. For instance, our previous study [45] demonstrated the simultaneous adsorption of cations and anions by a poly zwitterionic sulfobetaine ((*N*,*N*-dimethylacrylamido propyl) ammonium propane sulfonate) poly(DMAAPS) gel. However, in a KI solution, the adsorption capacity of the poly(DMAAPS) gel was ~13.7% of the sulfonate content (100 mmol/L) of the gel, which was relatively low. Therefore, this present study aimed to improve the ion adsorption capacity of the gel via co-polymerization between NIPAAM and DMAAPS in a specific molar ratio. The addition of the NIPAAM unit to poly zwitterionic sulfobetaine could reduce the inter- and intra-chain interactions and polymer chain entanglements of the polymer, potentially enhancing its ion adsorption.

In this study, poly(NIPAAM-*co*-DMAAPS) gels were used to adsorb ions in an aqueous solution. The co-polymerization between the thermosensitive monomer NIPAAM and zwitterionic betaine DMAAPS allowed the adsorption to be controlled by temperature. Notably, a previous study on the co-polymerization of sulfobetaine zwitterionic polymers and thermosensitive gels only emphasized the synthesis and properties of the gel or polymer independently [46]. To address the paucity of information concerning such polymers, this study aimed to elucidate the relationship between the adsorption capacity of the poly(NIPAAM-*co*-DMAAPS) gels in target solutions of chromium(III) nitrate (Cr(NO_3_)_3_) and other gel or polymer properties, such as the swelling ability, phase transition temperature, wettability, and visual analysis. The effects of the NIPAAM:DMAAPS ratio and temperature on the properties of each gel and polymer were investigated. Moreover, the ion adsorptions of the gels were compared with that of the zwitterionic polysulfobetaine homopolymer gel reported in our previous study [21].

## 2. Results and Discussion

### 2.1. Fourier-Transform Infrared Spectroscopy (FTIR) Analysis

**Figure 1:**
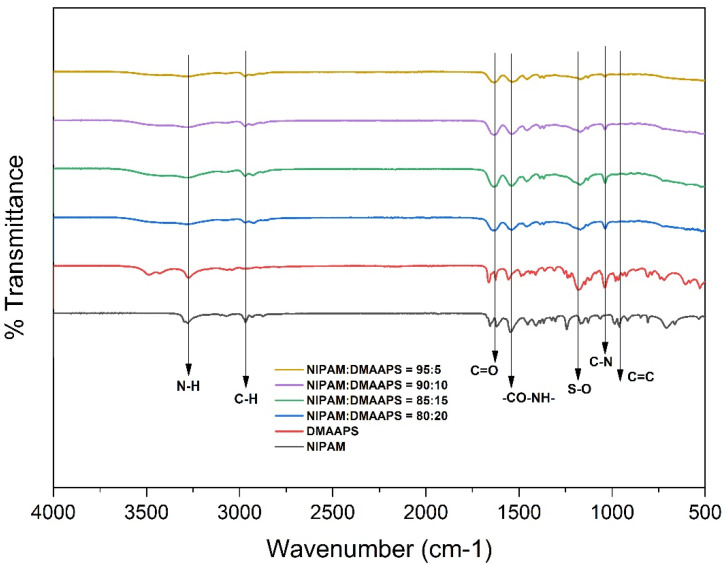
FTIR spectra of NIPAAM, DMAAPS, and poly(NIPAAM-*co*-DMAAPS) gels.

Figure 1 shows the FTIR spectra of poly(NIPAAM-*co*-DMAAPS) gels with concentration ratios of 100:0, 95:5, 90:10, 85:15, and 80:20. It was observed that the spectra changed due to the concentration variation. The difference in the functional groups of each constituent monomer and co-polymer was observed based on the presence of CH_2_=CH vinyl groups in the wavelength range of 900–1000 cm^−1^, which determines the success of the co-polymerization. In NIPAAM, the vinyl bond (CH_2_=CH) band was observed at 960 cm^−1^, whereas in DMAAPS, it was observed at 980 cm^−1^. However, in the spectrum of poly(NIPAAM-*co*-DMAAPS), this band was absent, indicating the successful co-polymerization of NIPAAM and DMAAPS. Additionally, with increasing DMAAPS concentration (5, 10, 15, 20, and then 100%), the intensity of the band corresponding to SO_3_^−^ (1170; 1181) also increased. All the functional group spectra in Figure 1 are in good agreement with our previous report [47] for poly(NIPAAM-*co*-DMAAPS), which shows that NIPAAM-*co*-DMAAPS is indeed the prepared co-polymer gel.

The wavenumber of each functional groups of poly(NIPAAM-co-DMAAPS) gels and its constituent monomer is shown in Table 1 which represent the FTIR spectra of Figure 1.

### 2.2. Proton Nuclear Magnetic Resonance (^1^H NMR) Analysis

**Figure 2:**
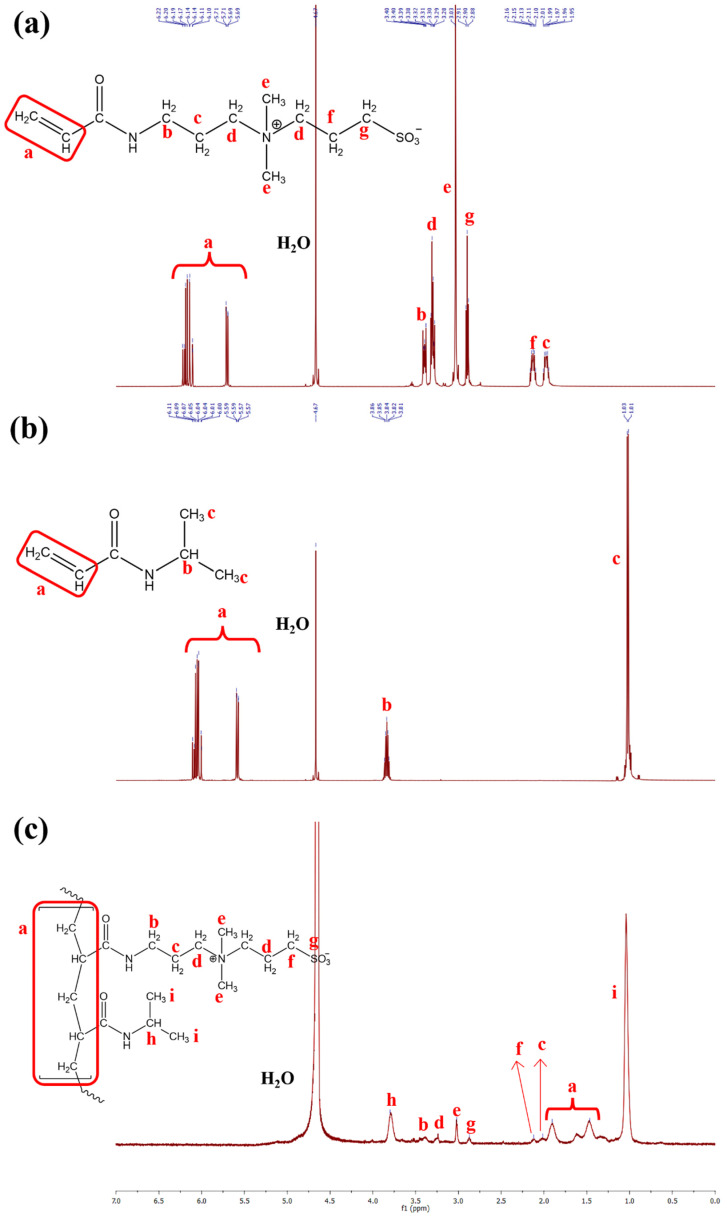
^1^H NMR spectra of DMAAPS (**a**) and NIPAAM (**b**) monomer and the resulting poly(DMAAPS-*co*-NIPAAM) (**c**). The NIPAAM:DMAAPS molar ratio is 95:5.

The chemical structures of the monomers and the resulting co-polymers were further confirmed using ^1^H NMR spectroscopy. The ^1^H NMR spectra of the NIPAAM and DMAAPS are displayed in Figure 2a,b, respectively. The two monomers were characterized by the presence of two peak signals between 5.5 and 6.5 ppm in the spectra, corresponding to the vinyl bond present in each monomer. Upon polymerization, these signals completely disappeared and peaks at 1.46 and 1.90 ppm (Figure 2c) appeared, indicating the head-to-tail polymerization required to assemble the polymeric structure of poly(NIPAAM-*co*-DMAAPS). 

### 2.3. Phase Transition Temperature Analysis

**Figure 3:**
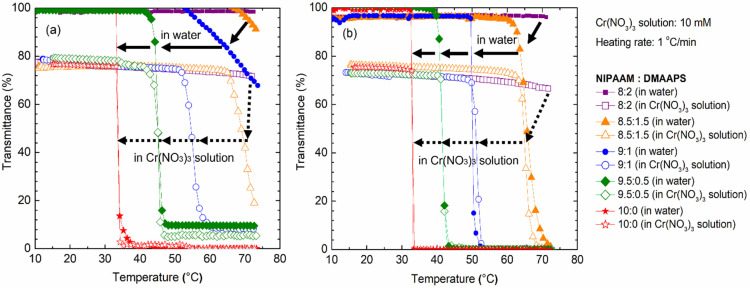
Phase transition temperature of poly(NIPAAM-*co*-DMAAPS) at polymer concentration of (**a**) 1 g/L and (**b**) 10 g/L. Heating rate = 1 °C/min in water and Cr(NO_3_)_3_ solution.

The LCST of a co-polymer indicates its phase transition in a solution. The LCST of a co-polymer can be determined by measuring the UV light transmittance of solution of the co-polymer under gradual heating. Figure 3 shows the temperature dependence of the transmittance of solutions of poly(NIPAAM-*co*-DMAAPS) in Cr(NO_3_)_3_ or water with various polymer concentrations (1 and 10 g/L) and monomer ratios. Subsequently, it was observed that increasing the NIPAAM ratio in the co-polymer resulted in the shift of LCST to lower temperatures, regardless of the co-polymer concentration. For H_2_O charged with 1 g/L co-polymer, the low NIPAAM ratios of 80:20 and 85:15 resulted in a light transmittance of approximately 100% throughout the given temperature range, revealing that the LCST is greater than 70 °C. The LCST was detected by the gradual decrease in transmittance starting at 70 °C as the NIPAAM ratio was increased to 90:10. A gradual decrease in LCST then occurred at 45 °C as the ratio was increased to 95:5. Further increasing the NIPAAM ratio to 100:0 lowered the LCST to 33 °C. Furthermore, when Cr(NO_3_)_3_ was added to the solution, 100% transmittance could not be achieved even at a temperature as low as 30 °C. In this case, the initial transmittance through the solution started at 80%, which slightly decreased with temperature. In this ionic solution, the co-polymer with the 80:20 NIPAAM ratio exhibited no significant drop in transmittance throughout the applied temperature range, suggesting that the LCST is greater than 70 °C. As the ratio was increased to 85:15, 90:10, 95:5, and 100:0, the LCST decreased to approximately 68, 54, 45 and 33 °C, respectively. Notably, as the NIPAAM ratio in the co-polymer was very high, the LCSTs were no longer influenced by the presence of Cr^3+^ ions, as observed at NIPAAM ratios of 95:5 and 100:0. This decrease in LCST is in agreement with a previous report on the aqueous co-polymer of P(NIPAm-*co*Zw10%) solutions [25] and can be explained as a combination of several effects: changes in the water structure in the polymer hydration sheath and changes in the interactions between the polymer and the solvent due to the presence of metal ions/salts [25,48,49].

The changes in the LCST were examined when the solution was enriched with 10 g/L of co-polymer. Irrespective of the temperature, the co-polymer with the low NIPAAM ratio of 80:20 could maintain approximately 100% transmittance through H_2_O; a similar trend was observed for this polymer at a lower concentration (1 g/L). However, further increasing the NIPAAM content to 85:15, 90:10, 95:5, and then 100:0 significantly decreased the LCST to 65, 51, 41, and 33 °C, respectively. Moreover, the LCST of 10 g/L of poly(NIPAAM-*co*-DMAAPS) in an aqueous Cr^3+^ solution was studied. As expected, the ionic solution contributed to the reduced light transmittance of the polymer, even at very low temperatures. Similar to the ionic solution with 1 g/L of poly(NIPAAM-*co*-DMAAPS), the LCST for the co-polymer with an 80:20 NIPAAM ratio was unrecognizable throughout the applied temperature range. Furthermore, changing the NIPAAM ratio to 85:15, 90:10, 95:15, and then 100:0 gave LCSTs of 64, 50, 41, and 33 °C, respectively. These results reveal that, in solutions with higher co-polymer content, the presence of the Cr^3+^ ions does not affect the LCST. For pure poly(NIPAAM), Cr^3+^ ions and the co-polymer concentration did not influence the LCST, which remained at 33 °C. 

The decrease in the LCSTs with increasing NIPAAM concentrations in water and Cr(NO_3_)_3_ solutions can be explained by the dominant properties of NIPAAM. When the NIPAAM ratio is high, the hydrophobic interactions between the hydrophobic backbone and the isopropyl group become considerably stronger and change the conformation of the co-polymer. The LCSTs of the co-polymer in water were found to be higher than those of the co-polymer in Cr(NO_3_)_3_. This was attributed to the ionic interactions of the ions in the Cr(NO_3_)_3_ solution (Cr^3+^ and NO_3_^−^) with the charged groups of DMAAPS (SO_3_^−^ and N^+^). However, at low ion concentrations, the number of ions available to disrupt the interactions of the SO_3_^−^ and N^+^ groups in poly(DMAAPS) was restricted. Therefore, only partial dissociation occurred, inducing chain mobility that consequently enhanced the inter-chain interactions, resulting in the aggregation of the co-polymer, as indicated by the decrease in the LCST. 

### 2.4. Visual Analysis of Color Change

**Figure 4:**
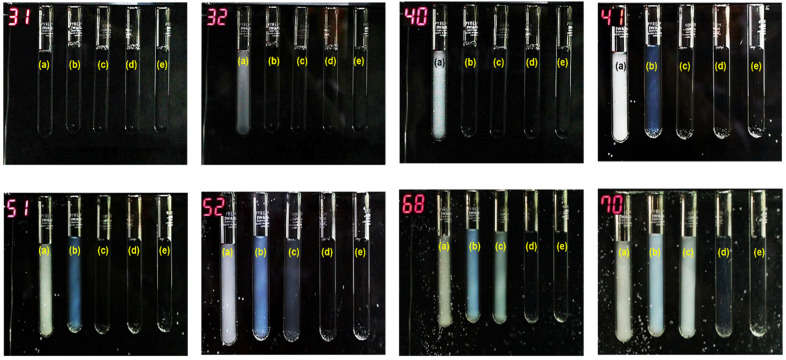
Photographs of H_2_O containing 1 g/L co-polymers with different monomer ratios (NIPAAM:DMAAPS): (**a**) 100:0, (**b**) 95:5, (**c**) 90:10, (**d**) 85:15, and (**e**) 80:20 at various temperatures.

Figure 4 shows the photographs of the 1 g/L aqueous poly(NIPAAM-*co*-DMAAPS) solutions with various monomer ratios at different temperatures. All the samples exhibited a clear appearance at 31 °C. As the temperature was increased to 32 °C, the poly(NIPAAM-*co*-DMAAPS) solution with a monomer ratio of 100:0 (tube a) exhibited a color/turbidity change. This is consistent with previous studies [25,50,51], which reported that poly(NIPAAM) homopolymer chains undergo reversible collapse or swelling in aqueous solutions above or below their transition temperatures/LCSTs, which is approximately 32–34 °C for the co-polymer in the present study. Furthermore, the solutions with the NIPAAM:DMAAPS ratios of 95:5 (tube b), 90:10 (tube c), and 85:15 (tube d) exhibited turbidity at 41, 52, and 70 °C, respectively. However, the solution in tube d was only slightly turbid at 70 °C. Moreover, no turbidity was observed at temperatures higher than 70 °C, suggesting the high miscibility of the co-polymer with the lowest NIPAAM:DMAAPS ratio. These observations suggest that the color change with temperature becomes less pronounced as the NIPAAM content decreases. This result correlates strongly with the decrease of LCST from 55 to 33 °C as the NIPAAM content increased from 90:10 to 100:0 (as shown in Figure 3a). The slight turbidity of tube d at 70 °C also corresponds with the minor decay in transmittance at this temperature. Similarly, a transmittance of approximately 100% can be achieved with the 80:20 NIPAAM ratio, which is in an agreement with the clear appearance of tube d. This observation suggests that decreasing the NIPAAM ratio gradually leads to the emergence of the DMAAPS character in the co-polymer, resulting in stronger interactions between the H_2_O molecules and DMAAPS moieties. Therefore, the co-polymer becomes more hydrophilic even at elevated temperature. These results are consistent with the observations made by Takeoka et al. [52]; in their study, the color of the thermosensitive pre-gel solution was tuned by controlling the amount of monomer.

### 2.5. Contact Angle Analysis

**Figure 5:**
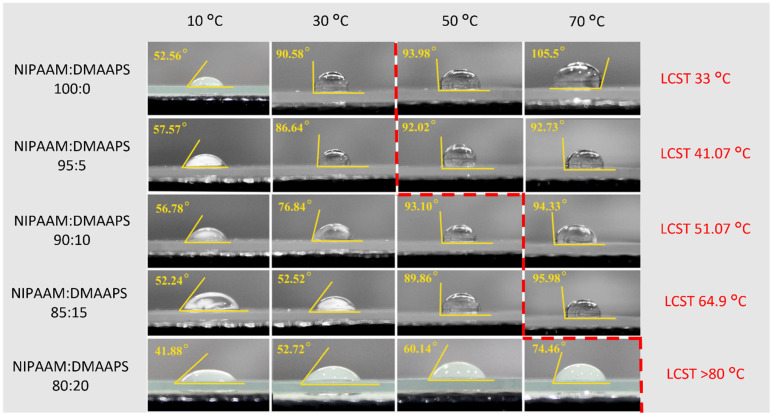
Contact angle analysis of co-polymers at different temperatures.

The static contact angle is the contact angle where the interfacial area between the liquid and solid phase does not change during the measurement. The measurement of static contact angles can be used to characterize the wettability or hydrophilic/hydrophobic properties of the poly(NIPAAM-*co*-DMAAPS) co-polymers. If the contact angle is less than 90°, the surface is hydrophilic, whereas if the angle is greater than 90°, the surface is hydrophobic. Furthermore, if the contact angle is between 150° and 180°, the surface is considered to be super-hydrophobic [53]. However, in several cases, the hydrophilic co-polymers have a contact angle greater than 90°. This occurs because the applied temperature during the measurement can affect the water droplet. In some cases, the water droplet temperatures are higher than the desired temperature. Figure 5 shows the contact angle created at the interface of poly(NIPAAM-*co*-DMAAPS) and the water droplet at various temperatures. For the co-polymer with a monomer ratio of 80:20, the contact angle remained <90° even at 70 °C. Moreover, this droplet exhibited turbidity, indicating that this co-polymer was hydrophilic at the tested temperatures. Under such conditions, the co-polymers were miscible, and the surface was hydrophilic. The hydrophobicity can be marked at 70 °C, as the NIPAAM ratio was set to 85:15. Upon increasing the NIPAAM ratio in the co-polymer from 90:10 to 100:0, the contact angle was >90° at 50 and 30 °C, respectively. Notably, the temperatures at which the hydrophobicity was first noticed were always higher than the LCSTs of the corresponding co-polymers. The hydrophobic character also contributed to the immiscibility of the co-polymer in water. Additionally, a red dashed line was created to denote the temperatures at which the hydrophobicity appeared. The co-polymers located on the left side are regarded as hydrophilic, and those on the right side are hydrophilic. Furthermore, the appearances of the droplets were observed at different temperatures. Figure 5 shows that the droplets turn clear when the contact angles are close to 90°. In contrast, cloudy droplets exhibit smaller contact angles. The color of the droplets might be influenced by their interaction with the co-polymer film. When the contact angle is small, the water droplets strongly interact with the co-polymer film beneath them. Furthermore, some fractions of the co-polymer may infiltrate the water droplet, and the concentration of this co-polymer within the droplet could increase over time, even in a small volume of water. At some point, the concentration of co-polymer exceeds a critical concentration for the phase transition, leading to the increased immiscibility. The enhanced immiscibility could suppress the transmittance, resulting in a droplet with a milky appearance. 

### 2.6. Cr^3+^ Ion Adsorption

**Figure 6:**
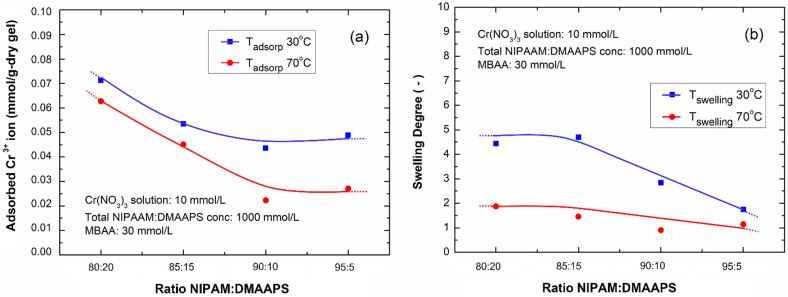
(**a**) Adsorption of Cr^3+^ by poly(NIPAAM-*co*-DMAAPS) gels at different temperatures. (**b**) Swelling degree of poly(NIPAAM-*co*-DMAAPS) gels at different temperatures.

The Cr^3+^ adsorptions of the poly(NIPAAM-*co*-DMAAPS)s gels are shown in Figure 6a. The adsorption was performed isothermally at 30 °C and 70 °C. A similar trend can be observed for both the temperatures, whereby the amount of adsorbed Cr^3+^ decreased with the increasing NIPAAM ratio from 80:20 to 90:10. Further enrichment of the NIPAAM ratio to 95:15 did not significantly improve the ion adsorption. This suppressed adsorption capacity is caused by the high NIPAAM content; at higher NIPAAM ratios, the fraction of DMAAPS in the co-polymer is much lower, leading to the depletion of the SO_3_^−^ and N^+^ sites that actively adsorb the Cr^3+^ ions. In addition to the monomer ratio, temperature can also affect the adsorption of Cr^3+^ ions. For the NIPAAM ratio of 80:20, a decrease in temperature from 70 to 30 °C enhanced the gel adsorption capacity for Cr^3+^ from 0.065 to 0.072 mmol/g dry-gel, respectively, which is almost twice that of pure DMAAPS for trivalent cations, as reported previously [21]. This improvement was observed for all the monomer ratios, suggesting that adsorption is more favorable at reduced temperature.

This observation is consistent with our previous study [21] in that, at high temperatures, the thermal motion weakens the interaction between the SO_3_^−^ and N^+^ groups of DMAAPS, owing to the charge dissociation. Moreover, the study also revealed that the highest adsorption capacity of poly(DMAAPS) was 0.039 mmol/g dry-gel. Accordingly, this result provides evidence that the co-polymerization of NIPAAM with DMAAPS could increase the molecular distance. This is because the NIPAAM units in the co-polymer filled the spaces between the DMAAPS units. Consequently, the interaction between SO_3_^−^ and N^+^ in the DMAAPS-charged groups is reduced. It eventually promotes the pairing of ions from the solution with the charged groups of the co-polymer, resulting in enhanced Cr^3+^ ion adsorption. In addition, these results also suggest that using a small concentration of DMAAPS in the co-polymerization is more favorable. With only 5–10% DMAAPS in the gel, similar ion adsorption to that of the poly(DMAAPS) homopolymer gel could be achieved. At this concentration, the maximum amount of Cr^3+^ ions adsorbed by a 10 mmol/L Cr(NO_3_)_3_ solution of 0.0454 mmol/g of gel was estimated to be ~58.49% of the sulfonate content in the gel. 

The swelling degrees of poly(NIPAAM-*co*-DMAAPS) gels with various monomer ratios are shown in Figure 6b. As can be observed, at 70 °C (red line), the NIPAAM concentration does not affect the swelling degrees of the gels. This behavior could be explained by the relatively much higher concentration of NIPAAM compared to DMAAPS, which caused the gel characteristics to be predominantly influenced by the properties of NIPAAM. Thus, at a higher temperature (70 °C), all gels tended to shrink, and the swelling degree was almost the same. In contrast, at a lower temperature (30 °C), the swelling degree gradually decreased with increasing NIPAAM concentration, and at 95% NIPAAM, the swelling degree was almost similar to that at 70 °C. This low swelling degree is also associated with the low ion adsorption by the gel (Figure 6a), which is due to its low DMAAPS content. Moreover, it was observed that the swelling degree at both temperatures was constant below 85% NIPAAM. This could be attributed to the impaired repulsion of DMAAPS chains by the adsorbed Cr^3+^ ions or the ionic crosslinking between SO_3_^−^ and Cr^3+^ ions.

The maximum amount of Cr^3+^ adsorbed by the poly(NIPAAM-*co*-DMAAPS)s gels is relatively high, i.e., 1852 mg/g, in comparison with the adsorption capacity of related adsorbents reported in previous studies, as listed in Table 2.

To clearly understand the relationship between the properties of the co-polymers and gels, the results of various analyses are summarized in Table 3 and Table 4.

It is clear that the operating conditions (i.e., temperature and monomer concentrations utilized in the preparation of the gels and co-polymers) significantly affects their properties. In the case of co-polymers, when the temperature was higher than the LCST, hydrophobicity was observed, as evidenced by their >90° contact angles. The co-polymer solutions were opaque and milky-white in appearance. This hydrophobic nature of the co-polymers also made the gels denser, as indicated by the lower swelling degree and lesser ionic interactions with the ions (Cr^3+^ and NO_3_^−^) in the Cr(NO_3_)_3_ solution. 

Moreover, when the temperature was lower than the LCST, the co-polymers exhibited hydrophilicity, as indicated by their <90° contact angle and the co-polymer solutions appeared transparent. The hydrophilic nature of co-polymers made the gels less dense, resulting in a higher degree of swelling. Consequently, more ionic interactions occurred between the gels and the ions in the Cr(NO_3_)_3_ solution, resulting in increased ion adsorption, particularly for the gels with high DMAAPS concentration. In future studies, the adsorption capacity, kinetics, and isotherm models of poly(NIPAAM-*co*-DMAAPS) gels can be investigated for various ions and solutions. Furthermore, the exact composition of each prepared gel can be further evaluated with respect to its correlation with the gel adsorption capacity.

## 3. Conclusions

Poly(NIPAAM-*co*-DMAAPS) co-polymers and gels were synthesized with various monomer ratios. The structures of the monomers and co-polymers were determined using FTIR and ^1^H NMR spectroscopies. The results of the phase transition temperature analysis showed that an increase in the DMAAPS concentration increased the LCST of the co-polymer, changing the co-polymer from hydrophobic to hydrophilic. The visual nature of the co-polymers in solution was also significantly affected by the monomer ratio. The co-polymers with high NIPAAM content exhibited a turbidity change in solution at higher temperatures and >90° contact angles. The ion adsorption tests confirmed that the co-polymerization of NIPAAM with DMAAPS increased the molecular distance and weakened the interactions between the DMAAPS-charged groups (SO_3_^−^ and N^+^), leading to enhanced ion adsorption. This observation suggests that using a lower concentration of DMAAPS via its co-polymerization with NIPAAM is more efficient than using a poly(DMAAPS) homopolymer gel. Nevertheless, the adsorption temperature must be adjusted to a value lower than the LCST of the co-polymer to achieve a higher ion adsorption capacity. Therefore, the operating temperature and DMAAPS concentration must be considered while designing co-polymers and gels for adsorption applications. In addition, future studies should elucidate the reversibility (adsorption/desorption) and ion selectivity of these co-polymers in a multi-ion solution at various temperatures and with a wider range of monomer concentrations.

## 4. Materials and Methods

### 4.1. Materials

NIPAAM and *N*,*N*-dimethylaminopropyl acrylamide (DMAPAA) were purchased from KJ Chemicals (Tokyo, Japan) and purified via *n*-hexane re-crystallization and vacuum distillation, respectively. 1,3-Propanesulfate (PS) and *N*,*N*′-methylenebisacrylamide (MBAA) were purchased from Tokyo Chemical Industry (Tokyo, Japan) and used as received without further purification. *N*,*N*,*N*′,*N*′-tetramethylethylenediamine (TEMED) and ammonium peroxide (APS) were purchased from Sigma–Aldrich (Burlington, MA, USA) and used as received without further purification. Cr(NO_3_)_3_ was purchased from Merck Millipore (Jakarta, Indonesia) and used as received without further purification.

### 4.2. Methods

#### 4.2.1. Monomer Synthesis

**Figure 7:**
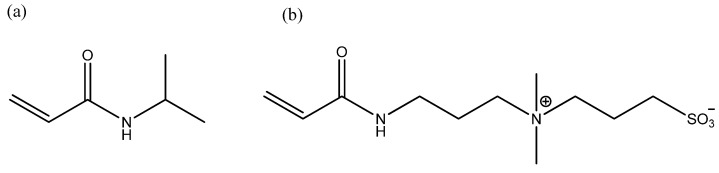
Chemical structure of (**a**) NIPAAM and (**b**) DMAAPS.

DMAAPS was synthesized from the ring-opening reaction of DMAPAA and PS [45]. A solution of PS (75 g) and acetonitrile (75 g) was added dropwise into the solution of DMAPAA (100 g) and acetonitrile (200 g) for 90 min under stirring at 30 °C. The mixture was continuously stirred for 16 h, and the solution was then left to stand for 2 d. The white crystals formed were then filtered, re-washed with acetone (500 mL), and oven-dried at 50 °C for 24 h. The chemical structures of NIPAAM and DMAAPS are shown in Figure 7. 

#### 4.2.2. Synthesis of Poly(NIPAAM-*co*-DMAAPS) Co-Polymers and Gels

Poly(NIPAAM-*co*-DMAAPS) co-polymers and gels were synthesized via free radical polymerization using a solution consisting of monomers (NIPAAM and DMAAPS), an initiator (APS), an accelerator (TEMED), and water. The NIPAAM:DMAAPS molar ratios were fixed at 100:0, 95:5, 90:10, 85:15, and 80:20. Typically, NIPAAM, DMAAPS, and TEMED (2 mol/L) were dissolved in distilled water to a total volume of 100 mL. This solution was then introduced into a three-neck separable flask and sealed. The solution was purged using ultra-high-purity nitrogen gas for 10 min to remove dissolved oxygen. Then, a similarly purged APS solution (2 mmol/L, 20 mL) was added to the reaction mixture. The polymerization reaction proceeded at 10 °C under continuous nitrogen flow and stirring for 6 h. The polymer was then separated and washed with distilled water through membrane dialysis with a molecular weight cut off of 12,000–14,000 (CelluSep T3, Membrane Filtration Products, Seguin, TX, USA) for one week. Poly(NIPAAM-*co*-DMAAPS) gels were synthesized using the same method, along with the addition of MBAA (30 mmol/L) as a crosslinker, in the same manner as outlined in previous studies [47,67]. The gel was washed with distilled water for 7 d. The water was changed every 12 h. The gel was dried on a Teflon paper placed in a Petri dish covered with a plastic film with small holes to decrease the gel drying rate. After drying, the gel was crushed into a 90-mesh size and used for the adsorption tests. 

#### 4.2.3. Characterization

FTIR spectroscopy (Nicolet IS10, Thermo Fisher Scientific, Waltham, MA. USA) was performed in the wavenumber range of 4000–500 cm^−1^ to identify the functional groups of NIPAAM, DMAAPS, and poly(NIPAAM-*co*-DMAAPS) gel. ^1^H NMR spectroscopy (MR400 DD2, Agilent, Santa Clara, CA, USA) was used to elucidate the chemical structure of poly(NIPAAM-*co*-DMAAPS) gel. The samples (20 mg) were dissolved in D_2_O solvent (1 mL).

The transition temperature of the samples was analyzed by observing the transmittance changes using UV–vis spectroscopy (V-630, Jasco Corp, Tokyo, Japan) with temperature control. The transmittance changes as a function of temperature were measured at a wavelength of 600 nm. The LCST can be determined at a temperature where the transmittance value is 50%. The LCST was obtained by heating the sample from 10 °C to 70 °C to observe changes in the sample transparency. Sample solutions (1 g/L) were placed in a cuvette equipped with a heater coil and heated at a rate of 1 °C/min. The transition temperature was also observed visually as the solutions changed from transparent to milky white.

Contact angle analysis was used to determine the hydrophobic/hydrophilic properties of the co-polymer. A sample solution (5 g/L) was dropped on a glass slide, uniformly spread by a spin coater, and then dried under vacuum at 50 °C for 30 min. Contact angle measurements were taken 60 s after water (~0.05 mL) was dropped onto the dried co-polymer surface to allow the droplet to stabilize. The internal contact angle was obtained from the line contact angle of the liquid–gas interface using co-polymer surface as the baseline. The visual analysis aimed to visually observe the phase transition of the co-polymer solution, as shown by the change in solution turbidity. If the solution was transparent, the component was completely miscible. Conversely, if the solution was milky white, the component was deemed immiscible. Images for contact angle and visual analyses were acquired using a Canon EOS 700D camera (50 mm lens, aperture f/8, shutter speed 1/500 s, ISO 200).

Atomic absorption spectroscopy (AAS) (Model 210 VGP, Buck Scientific, USA) was used to measure the ion concentrations before and after the adsorption by the gel in the target solution. The adsorption experiment was carried out by placing the dry-gel (1 g) in a glass bottle containing 20 mL of a 10 mmol/L Cr(NO_3_)_3_ solution. The bottle was put in a water bath heated to the desired temperature, and the gel-containing solution was then stirred for 12 h to allow the gel to achieve adsorption equilibrium. To determine the concentration of the cation (Cr^3+^) in the solution after adsorption, the gels were removed from the solution and filtered following centrifugation at 3000 rpm for 10 min.

In addition, the swelling degrees of the gels were determined using a 10 mmol/L Cr(NO_3_)_3_ solution at various temperatures to achieve equilibrium swelling for 24 h using a cathetometer, as reported previously [19]. The swelling degree is defined as the ratio of the swollen gel diameter to the dry-gel diameter.

## Figures and Tables

**Table 1:** Functional groups observed in FTIR spectra of NIPAAM, DMAAPS, and poly(NIPAAM-*co*-DMAAPS) gels.

No.	Functional Groups	Wavenumber (cm^−1^)					
		NIPAAM	DMAAPS	NIPAAM:DMAAPS = 80:20	NIPAAM:DMAAPS = 85:15	NIPAAM:DMAAPS = 90:10	NIPAAM:DMAAPS = 95:5
1	C=C	960	980	-	-	-	-
2	CO-NH	1547	1556	1540	1540	1537	1538
3	C-H	2968	2974	2934	2970	2970	2970
4	C-N	1061	1038	1036	1036	1037	1037
5	C=O	1620	1625	1634	1635	1632	1634
6	N-H	3279	3273	3276	3280	3281	3289
7	S-O	-	1181	1170	1170	1170	1170

**Table 2:** Comparison of different adsorbents toward the Cr^3+^ ions ^1^.

Adsorbent	Surface Area (m^2^/g)	Adsorption Capacity (mg/g)	Temperature (°C)	pH	Concentration (mg/L)	Ref.
MWCNTs	-	24.9	-	7	2–10	[54]
CM-BT	-	20.90	30	5.5	2	[55]
Nano-ZrO_2_-Glu-CMC	24.13	58.2	25	7	-	[56]
RPG	-	39.86	25	8	100	[57]
Spheroidal cellulose adsorbent containing the carboxyl anionic group	-	72.6	25	4	1920	[58]
PW	15.12	97.23	50	3.43	40	[59]
P-SBA-15	701	63.6	30	4	100	[60]
ZrO_2_-MMT	-	172.41	50	7–8	30	[61]
CP-Fe-Mn	89.3853	19.92	30	4.30	100	[62]
TF-SCMNPs	-	1.1	25	10	400	[63]
PVP/SiO_2_	873.62	97.7	30	7	100	[4]
NiO-MgO SBNs	48	209.5	25	5.5	50–400	[64]
Bagasse fly ash	480	2.48	25	6	30	[65]
Amberlite IRN-77 cation exchange resin	-	46.95	25	4,8	100	[66]
poly(NIPAAM-*co*-DMAAPS)	-	185.2	30	7	330	This study

^1^ Abbreviations. MWCNTs: multi-wall carbon nanotubes. CM-BT: immobilizing bayberry tannin (BT, a typical natural polyphenol) onto chitosan microfiber (CM). Nano-ZrO_2_-Glu-CMC: crosslinking of nanolayer carboxymethyl cellulose (CMC) onto the surface of nano-zirconium oxide (Nano-ZrO_2_) using glutaraldehyde. RPG: brown macroalga *Padina gymnospora.* PW: phosphate mine waste. P-SBA-15: phosphoric acid—modified. ZrO_2_-MMT: zirconium dioxide-loaded montmorillonite composites. CP-Fe-Mn: MnO_2_-modified magnetic bio-char. TF-SCMNPs: thiol-functionalized mesoporous silica-coated magnetite nanoparticles. PVP/SiO_2_: amino (-NH_2_) functionalized mesoporous polyvinyl pyrrolidone composite nanofiber membranes. NiO-MgO SBNs: nickel and magnesium oxides embedded into silica.

**Table 3:** Analysis results for co-polymers (phase transition temperature, contact angle, visual) and gels (swelling degree and ion adsorption) with various monomer ratios at 30 °C.

Molar Ratios of NIPAAM and DMAAPS	LCST (°C)	Temperature (30 °C)			
		Contact Angle (°)	Visual	Swelling Degree (-)	Ion Adsorption (mmol/g dry-gel)
95:05:00	41.072	86.64	hydrophilic,	1.75	0.0489
			transparent		
90:10:00	51.07	76.84	hydrophilic,	2.844	0.0454
			transparent		
85:15:00	64.904	52.52	hydrophilic,	4.695	0.0555
			transparent		
80:20:00	>80	52.72	hydrophilic,	4.444	0.0712
			transparent		

**Table 4:** Analysis results for co-polymers (phase transition temperature, contact angle, visual) and gels (swelling degree and ion adsorption) with various monomer ratios at 70 °C.

Molar Ratios of NIPAAM and DMAAPS	NIPAAM Concentration (mmol/L)	LCST (°C)	Temperature (70 °C)			
			Contact Angle (°)	Visual	Swelling Degree (-)	Ion Adsorption (mmol/g dry-gel)
95:05:00	950	41.072	92.73	hydrophobic,	1.143	0.027
				milky white		
90:10:00	900	51.07	94.33	hydrophobic,	0.903	0.0223
				milky white		
85:15:00	850	64.904	95.98	hydrophobic,	1.455	0.045
				opaque		
80:20:00	800	>80	74.46	hydrophilic,	1.76	0.0627
				transparent		

## Data Availability

Data are contained within the article.
